# Factors associated with terminated pregnancies in Sri Lanka: A case study of the Sri Lankan Demographic and health survey (DHS) 2016

**DOI:** 10.1371/journal.pone.0298639

**Published:** 2024-02-23

**Authors:** Kaludura Anupama Seuwandi Thabrew, Ranawaka Arachchige Chathuri Saranga Ranawaka, Senaratne Ranamukhaarachchi

**Affiliations:** 1 Faculty of Science, Sri Lanka Technological Campus, Padukka, Sri Lanka; 2 Faculty of Engineering and Technology, Sri Lanka Technological Campus, Padukka, Sri Lanka; University of KwaZulu-Natal College of Health Sciences, SOUTH AFRICA

## Abstract

Pregnancy termination is considered to have adverse effects on women’s health and to have created financial, economic, and social problems in their lives. This study aimed to identify factors associated with pregnancy termination in Sri Lanka. The study used 2016 Sri Lanka Demographic and Health Survey (DHS) data of 16,323 ever-married women aged 15–49, who were clustered in selected enumerated areas. A binary logistic random intercept multilevel model was fitted to find the association between pregnancy termination and the predictor variables in this study. The overall pregnancy termination rate among Sri Lankan women was 16.14%. Increasing age of women was found to be associated with increasing odds of pregnancy termination. Women who were overweight or obese had higher odds of pregnancy termination, with 14% and 36%, respectively, compared to women with a normal weight. With increasing parity, the likelihood of pregnancy termination decreased. Women who used contraceptives had a 24% higher likelihood of pregnancy termination than those who refrained from using them. Cohabiting women had a 57% higher chance of pregnancy termination. Working women had 15% higher odds than unemployed women. Women who experienced domestic violence had a 14% higher odds of pregnancy termination than those who did not. Women from the Northern, Eastern, and North Central provinces had a lower likelihood of pregnancy termination compared to those from the Western province. Women in the urban sector were more likely to terminate their pregnancy than those in the estate sector. Further, women residing in households where indoor smoking was permitted had a 13% greater chance of ending their pregnancy compared to non-smoking households. The study highlights the importance of restructuring education related to health and well-being, family planning, and work-life balancing for both women and their partners, and developing and implementing or strengthening policies and laws related to mitigating pregnancy termination including domestic violence for women.

## Introduction

A pregnancy can end with live birth or termination. Whenever a pregnancy is terminated due to a miscarriage, induced abortion, or stillbirth, it is referred as a terminated pregnancy [1]. The term "miscarriage", also known as "spontaneous abortion", refers to the loss of pregnancy before 20 weeks [2]. A stillbirth is the loss of pregnancy after 20 weeks from the time of commencement of pregnancy [3]. An induced abortion is a termination of pregnancy that is brought about through external interventions. In this study, when a woman experienced a pregnancy that ends in a miscarriage, induced abortion, or a stillbirth, it was considered a termination of pregnancy. Different countries have varying laws and perspectives regarding induced abortions. In some countries where there are legal restrictions as safe methods are not accessible, and individuals may turn to unsafe methods, which can harm maternal health and even result in death [4].

Ending a pregnancy, regardless of the method used, can have adverse effects on a woman’s mental and physical well-being, causing conditions like depression, anxiety, and concerns about fertility [5]. Additionally, it can give rise to challenges in the family, financial constraints, and generate a sense of shame, stigma, and societal disapproval [1, 6].

It is estimated that the annual number of miscarriages and induced abortions that occur globally is 23 million and 73 million, respectively [7, 8]. In 2019, a total of 1.9 million stillbirths occurred worldwide [7, 9, 10]. Additionally, most miscarriages and stillbirths are the highest in South Asian countries [11, 12] and 97% of all unsafe abortions occur in developing countries [13]. Due to these alarming statistics, terminated pregnancy is considered a major issue in maternal and child health globally [14].

In Sri Lanka, a South Asian country, the rate of stillbirths was 6.9 per 1000 births in 2021, and a national survey conducted in 1999 reported an abortion rate of 45 per 1000 women aged between 15 and 49 years [15, 16]. These numbers highlight the urgent need for immediate action as they represent the potential loss of future citizens, which is a national disaster.

Like many other nations, Sri Lanka also grapples with a substantial number of women in their reproductive years experiencing unwanted pregnancies, with a growing percentage resorting to abortion as a mean to avoid childbirth [17]. Furthermore, induced abortion is a crime in Sri Lanka, unless the mother’s life is at risk due to the pregnancy [18].

Several studies have been conducted to identify risk factors for abortions, miscarriages, and stillbirths in Sri Lanka [19–23]. Due to small sample sizes and the samples not representing the general population, the results obtained from these studies may have had low power and compromise the inferences that can be drawn from them.

Inadequate levels of education and employment in lower-skilled positions were notable contributors to the heightened risk of unsafe abortion. Further, a woman’s marital status, her decision-making regarding family size, and the gender composition of her existing children contribute to her vulnerability to unsafe abortions [21, 24, 25]. Furthermore, attitudes of abortion were found to be influenced by additional factors, including ethnicity, religion, age, the number of children a family has, individual exposure, and personal experiences related to the issue [26–31].

The occurrence of induced abortions was observed to be infrequent among individuals who were unmarried, students, and among adolescent women [32]. Further the knowledge of the illegal status of abortion was more prevalent through media channels. Seeking assistance, particularly from non-medical sources like spouses, friends, neighbors, and family increased the risk of undergoing an unsafe abortion [24].

Women aged over 35 years, face a heightened risk of miscarriage in their first three months of pregnancy, with obesity and unmanaged diabetes recognized as direct contributors to this risk [33]. Further, various maternal factors, including thrombophilia, antiphospholipid antibody syndrome, extreme weight, and hypertension are known to heighten the risk of miscarriage [34]. Other identified risk factors include cigarette smoking, excessive caffeine consumption, trauma, and malnutrition [34]. Furthermore, maternal age, elevated BMI, and a prior history of miscarriages have also been recognized as risk factors for experiencing miscarriages [35].

The most frequent maternal factors identified in the stillbirth of population encompass maternal age, educational attainment, employment status, chronic hypertension or pregnancy-induced hypertension, chronic diabetes mellitus or gestational diabetes, weight status (underweight, overweight, or obesity), null parity, and the presence of anemia [36]. Additionally, the factors linked to stillbirth include smoking during late pregnancy, having no previous pregnancies, using assisted reproductive technologies, alcohol and drug use, a history of prior stillbirth, experiencing social disadvantage, and belonging to various ethnic backgrounds [20, 37].

The Demographic and Health Survey (DHS) is a survey implemented by Inner City Fund (ICF) International that collects, analyzes, and disseminates accurate and nationally representative data. These surveys are conducted in many developing countries around the world, including Sri Lanka [38]. Although the DHS is a reliable source of nationally representative data, little to no research has been done on terminated pregnancies in Sri Lanka using the DHS data set.

The aim of this study is to contribute to the existing literature on terminated pregnancies in the Sri Lankan context by identifying factors associated with terminated pregnancies at the individual and community levels using data from the Sri Lanka DHS in 2016. Furthermore, this study will help identifying necessary interventions for reducing terminated pregnancy rates in Sri Lanka, which will benefit decision-makers, health authorities, and policymakers identifying solutions related to maternal and child health.

## Materials and methods

### Data source, sampling, and data collection

The study used secondary data obtained from the most recent DHS conducted in Sri Lanka in 2016 by the Department of Census and Statistics (DCS). The sample design of the 2016 DHS followed a two-stage stratified sampling design. In the initial phase of the design, 2,500 census blocks/ clusters were chosen as the primary sampling units (PSUs). Within these designated PSUs, 10–12 housing units were selected as the secondary sampling units (SSUs). A total of 18,302 ever-married women between the ages of 15–49 in the selected census blocks were interviewed using a questionnaire [38]. Thus, individuals were nested within selected census blocks, which, by the sample design, were also nested within sectors and districts, adding a clustering effect to the collected data. After removing irrelevant and unrealistic responses, the study sample was finalized with 16,323 respondents in 2476 clusters.

### Ethics approval

This study involves a secondary analysis of the data from the 2016 DHS Sri Lanka survey, which was obtained from the DCS Sri Lanka. Permission and the ethical approval for the study was obtained from the DCS Sri Lanka. Given that the research relied on pre-existing data, securing consent from the participants was deemed unnecessary. All protocols adhered to the relevant guidelines and regulations [39].

### Study variables

#### Response variable

Data on past miscarriages, stillbirths, and abortions were collected by interviewing the women who qualified to respond to the question "Have you ever had a pregnancy that ended in a miscarriage, abortion, or stillbirth?" in the questionnaire. The response options were "Yes" or "No". Therefore, the variable "Pregnancy Termination Status” was used as the response variable in the study and was coded as 0 for "Yes" and 1 for "No".

#### Explanatory variables

The explanatory variables used in the study, including individual-level and community-level factors are shown in Table 1. These variables were selected based on their theoretical relevance, practical significance, and availability in the data set.

**Table 1:** Details of explanatory variables.

Variable	Code
***Individual Level***	
Woman’s Age (Years)	1–15 to 24
	2–25 to 34
	3–35 to 49
Woman’s BMI^a/^	1-Underweight
	2-Normal Weight
	3-Overweight
	4-Obese
Highest educational level	1-Never attended school
	2-Pre-school or Primary
	3-Secondary
	4-Higher
Parity	0
	1
	2
	3 or more
Use of any contraceptive method	0-No
	1-Yes
Married/ Living with a man	1-Married
	2-Living with a man
	3-Separated/ Divorced/ Widowed
Woman’s Age at first union (Years)	1- Less than 15
	2–15 to 24
	3–25 to 34
	4–35 to 49
Woman’s working status	1-Yes
	2-No
Encountered any domestic violence	0-No
	1-Yes
Smoking allowed inside the home	1-Yes
	2-No
Wealth index quintile	1-Lowest
	2-Second
	3-Middle
	4-Fourth
	5-Highest
***Community Level***	
Region	1-Western Province
	2-Central Province
	3-Southern Province
	4-Northern Province
	5-Eastern Province
	6-Northwestern Province
	7-North Central Province
	8-Uva Province
	9-Sabaragamuwa Province
Locality	1-Urban
	2-Rural
	3-Estate

^a/^ BMI—Body Mass Index

### Statistical analysis

The prevalence of pregnancy terminations was analyzed using univariate and bivariate analysis for the descriptive part of the variables. Then, the multicollinearity among the explanatory variables was checked using the Variance Inflation Factor (VIF) before fitting the model [40]. Afterward, a binary logistic random intercept multilevel model was employed due to the hierarchical nature of the data set, which consisted of fixed and random effects. The fixed effects show the association of the explanatory variables with the response variable, while the random effect, a measure of random variation, was assessed using the Intra-Cluster Correlation (ICC) [41]. Goodness of fit of the models was assessed using likelihood ratio test.

The statistical analysis for this study was conducted using SAS on Demand for Academics. SAS is a powerful and widely used software suite for data analysis, management, and reporting. It has been used in various industries, including healthcare, finance, and academia, for over four decades. All statistical tests were performed at a 5% level of significance.

## Results

### Baseline characteristics

The distribution of independent categorical variables across pregnancy termination statuses is presented in Table 2. The overall percentage of women who have ever had a pregnancy that ended in a miscarriage, abortion, or stillbirth accounted for 16.14% in the study sample. The majority of the 16,323 eve-married women in the sample lived in rural areas (78.75%). Of the women, 66.55% had education up to secondary level and more than half (57.72%) was between 35 and 49 years old. The majority of women (66.53%) was between 15 and 24 years old when they first unionized with a male. More than one-third of the women had a parity of two (36.78%) and 45.9% reported a normal Body Mass Index (BMI). Regarding marital status, 90.67% of women was married and most reported not experiencing any domestic violence from their husband or partner. Of the women, 67.85% was unemployed. The majority of women was from the Western Province (19.46%) and lived in households with a low wealth index quintile (23.41%). Moreover, according to the recorded data, 84.41% of women lived in households where smoking indoors was not allowed.

**Table 2:** Distribution of independent categorical variables across pregnancy termination status.

Variables	Overall (%)	Pregnancy Not Terminated	Pregnancy Terminated
	(n=16323)	(n= 13676)	(n= 2634)
	N (%)	N (%)	N (%)
**Individual Level**			
Highest educational level			
Never attended school	264 (1.62)	222 (84.09)	42 (15.91)
Pre-school or Primary	1 262 (7.73)	1 100 (87.16)	162 (12.84)
Secondary	10 863 (66.55)	9 071 (83.50)	1 792 (16.50)
Higher	3 934 (24.10)	3 296 (83.78)	638 (16.22)
Woman’s Age (Years)			
15–24	1 295 (7.93)	1 176 (90.81)	119 (9.19)
25–34	5 606 (34.34)	4 840 (86.34)	766 (13.66)
35–49	9 422 (57.72)	7 673 (81.44)	1 749 (18.56)
Woman’s BMI			
Underweight	1 503 (9.21)	1 298 (86.36)	205 (13.64)
Normal Weight	7 492 (45.90)	6 381 (85.17)	1 111 (14.83)
Overweight	5 192 (31.81)	4 297 (82.76)	895 (17.24)
Obese	2 136 (13.09)	1 713 (80.20)	423 (19.80)
Woman’s age at first union (Years)			
<15	254 (1.56)	216 (85.04)	38 (14.96)
15–24	10 859 (66.53)	9 183 (84.57)	1 676 (15.43)
25–34	4 925 (30.17)	4 057 (82.38)	868 (17.62)
35–49	285 (1.75)	233 (81.75)	52 (18.25)
Parity			
0	1 494 (9.15)	1 254 (83.94)	240 (16.06)
1	3 852 (23.60)	3 268 (84.84)	584 (15.16)
2	6 003 (36.78)	4 992 (83.16)	1 011 (16.84)
3 or more	4 974 (30.47)	4 175 (83.94)	799 (16.06)
Use of contraceptive method			
No	2 482 (15.21)	2 146 (86.46)	336 (13.54)
Yes	13 841 (84.79)	11 543 (83.40)	2 298 (16.60)
Married/ Living with a man			
Married	14 800 (90.67)	12 383 (83.67)	2 417 (16.33)
Living with a man	578 (3.54)	476 (82.35)	102 (17.65)
Separated/ Divorced/ Widowed	945 (5.79)	830 (87.83)	115 (12.17)
Woman’s working status			
Yes	5 248 (32.15)	4 310 (82.13)	938 (17.87)
No	11 075 (67.85)	9 379 (84.69)	1 696 (15.31)
Encountered any domestic violence			
No	13 137 (80.48)	11 038 (84.02)	2 099 (15.98)
Yes	3 186 (19.52)	2 651 (83.21)	535 (16.79)
Smoking allowed inside the home			
Yes	2 544 (15.59)	2 099 (82.51)	445 (17.49)
No	13 779 (84.41)	11 590 (84.11)	2 189 (15.89)
Wealth index quintile			
Lowest	3 822 (23.41)	3 296 (86.24)	526 (13.76)
Second	3 309 (20.27)	2 811 (84.95)	498 (15.05)
Middle	3 205 (19.63)	2 663 (83.09)	542 (16.91)
Fourth	3 126 (19.15)	2 592 (82.92)	534 (17.08)
Highest	2 861 (17.53)	2 327 (81.34)	534 (18.66)
**Community Level**			
Region			
Western Province	3 177 (19.46)	2 580 (81.21)	597 (18.79)
Central Province	1 965 (12.04)	1 600 (81.42)	365 (18.58)
Southern Province	1 818 (11.14)	1 485 (81.68)	333 (18.32)
Northern Province	2 003 (12.27)	1 779 (88.82)	224 (11.18)
Eastern Province	1 696 (10.39)	1 461 (86.14)	235 (13.86)
Northwestern Province	1 892 (11.59)	1 587 (83.88)	305 (16.12)
North Central Province	1 169 (7.16)	1 001 (85.63)	168 (14.37)
Uva Province	1 134 (6.95)	949 (83.69)	185 (16.31)
Sabaragamuwa Province	1 469 (9.00)	1 247 (84.89)	222 (15.11)
Locality			
Urban	2 571 (15.75)	2 131 (82.89)	440 (17.11)
Rural	12 854 (78.75)	10 771 (83.79)	2 083 (16.21)
Estate	898 (5.50)	787 (87.64)	111 (12.36)

### Results of binary logistic random intercept multilevel model

The mean VIF was 1.25, indicating the absence of high multicollinearity between the explanatory variables. Table 3 presents the results of the fitted binary logistic random intercept multilevel models. The goodness of fit of the models was evaluated using the Likelihood Ratio (LR) test. The interpretation of the fixed effects was based on the model with the lowest -2 Log Likelihood, which was Model 3.

**Table 3:** Individual and community level factors associated with pregnancy termination (n=16323).

Variable	Model 1		Model 2		Model 3	
	AdjOR^a/^	CI_95_	AdjOR	CI_95_	AdjOR	CI_95_
**Fixed Effects**						
***Individual level***						
Woman’s Age (Years)						
15–24			1.00		1.00	
25–34			1.73***	(1.39, 2.15)	1.73***	(1.39, 2.15)
35–49			2.68***	(2.13, 3.37)	2.64***	(2.10, 3.31)
Woman’s BMI						
Underweight			1.00	(0.85, 1.19)	1.00**	(0.85, 1.18)
Normal weight			1.00		1.00	
Overweight range			1.14*	(1.03, 1.26)	1.14***	(1.04, 1.26)
Obese			1.36***	(1.19, 1.54)	1.36***	(1.20, 1.55)
Woman’s Age at first union (Years)						
<15			1.00		1.00	
15–24			0.95	(0.66, 1.35)	0.95	(0.66, 1.36)
25–34			0.94	(0.65, 1.36)	0.94	(0.65, 1.37)
35–49			0.85	(0.52, 1.38)	0.84	(0.52, 1.37)
Highest education level						
Never attended school			1.00		1.00	
Pre-school or Primary			0.77	(0.53, 1.12)	0.79	(0.54, 1.15)
Secondary			1.08	(0.76, 1.53)	1.10	(0.77, 1.56)
Higher			0.92	(0.64, 1.32)	0.94	(0.65, 1.36)
Parity						
0			1.00		1.00	
1			0.75**	(0.62, 0.90)	0.78**	(0.64, 0.94)
2			0.66***	(0.54, 0.80)	0.69**	(0.57, 0.84)
3 or more			0.58***	(0.47, 0.71)	0.62***	(0.50, 0.76)
Use of contraceptive method						
Yes			1.36***	(1.18, 1.58)	1.24**	(1.07, 1.45)
No			1.00		1.00	
Married/ Living with a man						
Married			1.52***	(1.24, 1.88)	1.51**	(1.22, 1.86)
Living with a man			1.64**	(1.21, 2.21)	1.57**	(1.16, 2.13)
Separated/ Divorced/ Widowed			1.00		1.00	
Woman’s working status						
Yes			1.16**	(1.06, 1.28)	1.15**	(1.05, 1.27)
No			1.00		1.00	
Encountered any domestic violence						
Yes			1.13*	(1.01, 1.26)	1.14*	(1.02, 1.28)
No			1.00		1.00	
Smoking allowed inside the home						
Yes			1.15*	(1.02, 1.29)	1.13*	(1.01, 1.28)
No			1.00		1.00	
Wealth index quintile						
Lowest			1.00		1.00	
Second			1.02	(0.89, 1.17)	0.92	(0.80, 1.07)
Middle			1.15*	(1.00, 1.33)	1.01	(0.87, 1.17)
Fourth			1.14	(0.98, 1.31)	0.97	(0.83, 1.14)
Highest			1.22*	(1.05, 1.43)	1.01	(0.85, 1.20)
***Community Level***						
Region						
Western Province					1.00	
Central Province					1.13	(0.96, 1.33)
Southern Province					1.02	(0.86, 1.19)
Northern Province					0.60***	(0.50, 0.73)
Eastern Province					0.80*	(0.67, 0.97)
Northwestern Province					0.86	(0.73, 1.01)
North Central Province					0.78*	(0.64, 0.95)
Uva Province					0.95	(0.78, 1.15)
Sabaragamuwa Province					0.84	(0.70, 1.01)
Locality						
Urban					1.00	
Rural					1.01	(0.89, 1.15)
Estate					0.70**	(0.53, 0.92)
**Random Effects**						
Cluster Variance (SE^b^)	0.13*** (0.04)		0.10** (0.04)		0.08* (0.04)	
ICC	0.04		0.03		0.02	
**Model Fit**						
-2 Log Likelihood	14413.54		14160.87		14110.70	
BIC^c^	14429.17		14364.04		14392.02	
AIC^d^	14417.54		14212.87		14182.70	

^a/^ AdjOR: Adjusted Odds Ratio, CI_95_—95 percent Confidence Interval;

^b/^ SE: Standard Error;

^c/^ BIC: Bayesian Information Criterion;

^d/^ AIC: Akaike Information Criterion;

^e/^ Level of significance:

* p < 0.05,

** p < 0.01,

*** p < 0.001

### Results of measures of associations

The complete model including both individual and community-level factors was found in Model 3 in Table 3. Individual factors such as woman’s age, BMI, parity, contraceptive use, marital status, employment status, exposure to domestic violence, and smoking status inside the house were found significant. Some levels of region and locality were also significant among the community-level factors.

The odds of pregnancy termination increased significantly with the increasing age of women. Women aged 35–49 years old [AOR = 2.64, 95% CI = (2.10, 3.31)] had approximately three times higher odds of pregnancy termination compared to those women aged 15–24 years old. Overweight [AOR = 1.14, 95% CI = (1.04, 1.26)] and obese [AOR = 1.36, 95% CI = (1.20, 1.55)] women had 14% and 36% higher odds of pregnancy termination, respectively, compared to women with normal weight. The odds of pregnancy termination decreased with the increasing number of children a woman had. Women with three or more children [AOR = 0.62, 95% CI = (0.50, 0.76)] had 38% lower odds of pregnancy termination compared to women with zero parity. Women who used contraceptives [AOR = 1.24, 95% CI = (1.07, 1.45)] had 24% higher odds of pregnancy termination compared to otherwise. Cohabiting women [AOR = 1.57, 95% CI = (1.16, 2.13)] had 57% higher odds of pregnancy termination compared to separated, divorced, or widowed women. Working women [AOR = 1.15, 95% CI = (1.05, 1.27)] had 15% higher odds of pregnancy termination compared to unemployed women. Women who faced domestic violence [AOR = 1.14, 95% CI = (1.02, 1.28)] had 14% higher odds of pregnancy termination compared to otherwise. Women from the Northern [AOR = 0.60, 95% CI = (0.50, 0.73)], Eastern [AOR = 0.80, 95% CI = (0.67, 0.97)], and North Central [AOR = 0.78, 95% CI = (0.64, 0.95)] provinces had a lesser likelihood of pregnancy termination compared to women from the Western province. Women in the estate sector [AOR = 0.70, 95% CI = (0.53, 0.92)] had 30% lower odds of pregnancy termination compared to women in the urban sector. Women who resided in houses where smoking was permitted indoors [AOR = 1.13, 95% CI = (1.01, 1.28)] had 13% higher odds of pregnancy termination compared to those who did not.

### Results of measures of variance

Model 1, the empty model, indicated a statistically significant variability in the odds of pregnancy terminations with a cluster variance of 13%. Further, the ICC of the empty model shows that 4% of the total variability in the pregnancy terminations in Sri Lanka was attributed to differences between clusters, leaving 96% of the variability to be accounted for by other unknown factors. Model 3, the full model, adjusted for individual and community-level factors, reduced the cluster variance to 0.08 (Standard Error (SE) = 0.04) which remained significant. This shows that 2% of the total variance of pregnancy terminations was attributed to clusters. In a multilevel study, an ICC equal to or greater than 2% indicates significant group-level variance and is considered a minimum precondition [42].

## Discussion

This study analyzed the association of women’s and community characteristics with pregnancy termination in Sri Lanka using data collected under the Sri Lanka DHS 2016. A binary logistic random intercept multilevel model was fitted to identify the factors related to pregnancy termination. The results showed that the woman’s age, BMI, parity, contraceptive use, marital status, working status, domestic violence encounter status, smoking status inside the house, region, and locality significantly explained the variations in pregnancy termination in Sri Lanka.

### Factors associated with pregnancy termination in Sri Lanka

The fitted binary logistic random-intercept multilevel model showed that the likelihood of pregnancy termination increased with increasing age of women, which is in agreement with previous studies [43, 44]. This increase in pregnancy termination is attributed to the aging reproductive system of women, leading to negative pregnancy outcomes that may necessitate or cause a pregnancy termination [45]. In the Sri Lankan context, induced abortions are illegal unless they are performed to save the life of the mother. However a study done in Sri Lanka found the older age as a determinant of induced abortion [22]. Moreover, local studies show that advanced maternal age was associated with adverse obstetric and perinatal outcomes including stillbirths and miscarriages [33, 35, 46].

The fitted binary logistic random intercept multilevel model showed that woman’s BMI was a significant factor associated with pregnancy termination. Women who did not have a normal weight on the BMI scale (18.5 kg/m^2^ to 24.9 kg/m^2^) [47] experienced significantly higher rates of pregnancy termination. Similar results on the association of pregnancy termination, mostly attributed to miscarriages and stillbirths, and BMI was observed in previous local and global studies [33, 35, 48–51]. The reasons behind the higher rates of pregnancy termination due to a higher BMI compared to normal weight are still unclear, but studies have found that factors such as high blood pressure, pre-gestational and gestational diabetes, and placental diseases or disorders are more likely to occur in overweight or obese women which can cause adverse pregnancy outcomes [52, 53]. In addition, underweight women experience infertility due to hormonal disturbances caused by eating disorders and lack of nutrients, and they may also experience miscarriages and sexual dysfunction [54]. Therefore, apart from miscarriages and stillbirths, induced abortion could be performed to save the mother’s life in cases where life threatening pregnancy complications occur. However, the association between induced abortion and not having a normal BMI needs further investigation.

The odds of a terminated pregnancy were high among women who had no children compared to those women who had children. Furthermore, the study observed lower odds of pregnancy termination with increasing parity, which is consistent with previous studies conducted in Sub-Saharan Africa (SSA) [55] and Ghana [56]. A study done in Sri Lanka found that nulliparous women have higher likelihood of giving stillbirths [36]. However, this aspect in terms of miscarriage and induced abortion has not been explored much in local literature and hence needs further investigation.

The likelihood of pregnancy termination was high among women who have used contraceptives, which aligns with the findings of prior studies [57, 58]. The use of contraceptive methods, especially oral contraceptive pills, can cause hormonal disruptions, and these disruptions have been found to increase the risk of pregnancy complications that can lead to terminations, such as stillbirths, miscarriages, or abortions [59]. Furthermore, a previous Sri Lankan study revealed that the practice of traditional contraception could lead to repeated abortions, and in that scenario women may consider induced abortion as a contraceptive method or back-up procedure for contraceptive failure [60].

This study found that, unlike the results of other studies [61, 62], married and cohabiting women had a higher risk of pregnancy termination compared to women who were not married or not living with their partner. Furthermore, a previous study conducted in Sri Lanka found that the susceptibility of unmarried women to induced abortion was not influenced by their social standing and highlights the societal stigma attached to ’unwed mothers’ in Sri Lanka, where cohabitation outside of marriage is not culturally accepted [21]. Also, evidence shows that it is married women, over the age of 30 years, with at least one or two (living) children (of a very young age), who often seek abortion services [63].

The binary logistic random intercept multilevel model showed that working women had a higher likelihood of pregnancy termination compared to non-working women. These findings are supported by research conducted in SSA [55]. Stress at work, long working hours, and a heavy physical workload significantly contribute to pregnancy terminations [64–66]. Previous studies in Sri Lanka show that, in recent decades, women have increasingly sought independence to actively contribute to both family finances and social responsibilities, much like their partners. This endeavor may be a probable factor contributing to the elevated risk of unsafe abortions [21]. Furthermore, the availability of job opportunities for women in the Middle-East, especially within the lower economic strata of the local labor market is cited as a factor leading to the choice of abortion as a method of ending pregnancies and preventing births [67].

The results of this study showed that the likelihood of pregnancy termination was high among women who lived in houses where smoking was allowed indoors. Women typically spend a lot of time at home during pregnancy and inhaling cigarette smoke, even if they do not smoke themselves, for an extended period can expose them to passive smoking. According to the World Health Organization (WHO), exposure to passive smoking can lead to adverse effects on pregnancy outcomes [68]. Previous studies have also shown that miscarriage, stillbirth, and abortion is significantly associated with passive smoking [69–73]. However, it would be worth conducting further studies on the association between pregnancy termination, including miscarriage, stillbirth, and induced abortion, and living in houses where indoor smoking allowed, as there has been a few or no detailed studies conducted in Sri Lanka on this matter.

The results of the fitted model show that women who experienced domestic violence have a significantly higher likelihood of terminating their pregnancies than those who have not experienced any domestic violence. These results are consistent with the findings of previous studies [74, 75]. A plausible reason for this could be that physical violence and accidental blows on a woman, both during and outside of pregnancy, can cause physical damage, especially to the lower abdomen region, and also result in mental distress which can lead to pregnancy complications and result in termination [75–77]. In a previous local study, it was shown that instances of domestic violence can result in induced abortions, as the woman may feel compelled to end the pregnancy due to it being unwanted or a consequence of non-consensual sexual engagement [78].

The results of the fitted model show that the odds of pregnancy terminations are significantly lower in regions with low population density, such as the Northern, Eastern, and North Central provinces, compared to that in the Western province, which has the highest population density [79]. Furthermore, the estate sector, which has the lowest density at the locality/sector level [79], has a lower likelihood of pregnancy terminations compared to the urban sector. These results may have been influenced by population density, as lower numbers of cases were reported in regions and localities with lower population density when compared to denser regions and localities. However, past research has indicated that districts with fewer urban areas and marginalized populations tend to have higher rates of unsafe abortions [80]. This warrants further investigations.

### Limitations of the study

The present study used only the 2016 DHS data set. Therefore, causal inference between the predictor variables and pregnancy termination was not considered. Although the study dataset has many variables that could be considered for this study, not all of them were included because the fitted model faced converging issues and hence, those variables were removed from the study. Furthermore, the survey used in data collection relied up on self-reported data, so there may be inaccurate or misleading information and as a result a recalled bias. Additionally, since induced abortion is illegal in Sri Lanka, unless women were granted permission to discuss it confidentially, many respondents who have undergone an induced abortion might not have admitted to it. This could have resulted in a probable underreporting of this issue, affecting the obtained results.

## Conclusions

This study identified factors associated with pregnancy termination in Sri Lanka. The results indicated that increasing age of women, being classified as either overweight or obese based on the BMI scale, exposure to passive smoking at home, use of contraceptive methods, being married or cohabiting, being employed, and experiencing domestic violence increase the odds of pregnancy termination. Moreover, having more children (increasing parity) is linked to a lower likelihood of pregnancy termination. Additionally, community-level variables like region and locality were identified as significant factors associated with pregnancy termination. These findings highlight the time to call for the improvement of education related to health and wellbeing, family planning, work-life balance for both women and their partners, and the implementation or strengthening of policies and laws related to domestic violence for women. Implementing these measures can ensure safe and positive pregnancy outcomes and ensuring the human capital in the future of Sri Lanka.

## Abbreviations

AOR: Adjusted Odds Ratio
BMI: Body Mass Index
CI: Confidence Interval
DCS: Department of Census and Statistics
DHS: Demographic and Health Survey
ICC: Intra-Cluster Correlation
ICF: Inner City Fund
LR: Likelihood Ratio
SE: Standard Error
SSA: Sub-Saharan Africa
VIF: Variance Inflation Factor
WHO: World Health Organization

## References

[1] MacQuarrie K, Winfrey W, Meijer-Irons J, Morse AR. Consistency of reporting of terminated pregnancies in DHS calendars (DHS Methodological Reports No. 25). Rockville, MD; 2018.

[2] Dugas C, Slane VH. Miscarriage. In: StatPearls [Internet] [Internet]. StatPearls Publishing; 2022. https://www.ncbi.nlm.nih.gov/books/NBK532992/

[3] Anon. What is Stillbirth? [Internet]. Centers for Disease Control and Prevention. 2022. https://www.cdc.gov/ncbddd/stillbirth/facts.html

[4] Megersa BS, Ojengbede OA, Deckert A, Fawole OI. Factors associated with induced abortion among women of reproductive age attending selected health facilities in Addis Ababa, Ethiopia: a case control study. BMC Womens Health. 2020;20(1):1–11. 32883263 10.1186/s12905-020-01023-4 PMC7469090

[5] Ojule JD, Ogu RN. Miscarriage and Maternal Health. In: Abduljabbar H, editor. Complications of Pregnancy [Internet]. Rijeka: IntechOpen; 2019. doi: 10.5772/intechopen.82117

[6] Anon. Stillbirth [Internet]. World Health Organization. 2020. https://www.who.int/health-topics/stillbirth

[7] Quenby S, Gallos ID, Dhillon-Smith RK, Podesek M, Stephenson MD, Fisher J, et al. Miscarriage matters: the epidemiological, physical, psychological, and economic costs of early pregnancy loss. Lancet. 2021 May 1;397(10285):1658–67. doi: 10.1016/S0140-6736(21)00682-6 33915094

[8] Bearak J, Popinchalk A, Ganatra B, Moller AB, Tunçalp Ö, Beavin C, et al. Unintended pregnancy and abortion by income, region, and the legal status of abortion: estimates from a comprehensive model for 1990–2019. Lancet Glob Heal. 2020 Sep 1;8(9):e1152–61. doi: 10.1016/S2214-109X(20)30315-6 32710833

[9] Ahinkorah BO. Intimate partner violence against adolescent girls and young women and its association with miscarriages, stillbirths and induced abortions in sub-Saharan Africa: evidence from demographic and health surveys. SSM-Population Heal. 2021;13:100730. doi: 10.1016/j.ssmph.2021.100730 33511264 PMC7815812

[10] Hug L, You D, Blencowe H, Mishra A, Wang Z, Fix MJ, et al. Global, regional, and national estimates and trends in stillbirths from 2000 to 2019: a systematic assessment. Lancet. 2021;398(10302):772–85. doi: 10.1016/S0140-6736(21)01112-0 34454675 PMC8417352

[11] Xue T, Guan T, Geng G, Zhang Q, Zhao Y, Zhu T. Estimation of pregnancy losses attributable to exposure to ambient fine particles in south Asia: an epidemiological case-control study. Lancet Planet Heal. 2021;5(1):e15–e24. doi: 10.1016/S2542-5196(20)30268-0 33421406

[12] Stone R, Palmer K, Wallace EM, Davey M-A, Hodges R, Davies-Tuck M. Fetal monitoring from 39 weeks’ gestation to identify South Asian-born women at risk of perinatal compromise: a retrospective cohort study. Sci Rep. 2021;11(1):1–7. 34857850 10.1038/s41598-021-02836-5 PMC8639724

[13] Anon. Abortion [Internet]. World Health Organization. 2021. https://www.who.int/news-room/fact-sheets/detail/abortion

[14] Ahinkorah BO, Seidu A-A, Ameyaw EK, Budu E, Bonsu F, Mwamba B. Beyond counting induced abortions, miscarriages and stillbirths to understanding their risk factors: analysis of the 2017 Ghana maternal health survey. BMC Pregnancy Childbirth. 2021;21(1):1–10. 33593319 10.1186/s12884-021-03633-8 PMC7885363

[15] Anon. National Statistics [Internet]. Family Health Bureau Ministry of Health Sri Lanka. 2022. https://fhb.health.gov.lk/index.php/en/statistics/statistics-2

[16] Kumar R. Abortion in Sri Lanka: The Double Standard. Am J Public Health. 2013 Mar;103(3):400. doi: 10.2105/AJPH.2012.301154 23327236 PMC3673519

[17] Ban DJ, Kim J, De Silva WI. Induced abortion in Sri Lanka: who goes to providers for pregnancy termination? J Biosoc Sci. 2002;34(3):303–15. doi: 10.1017/s0021932002003036 12117211

[18] Perera J, de Silva T, Gange H. Knowledge, behaviour and attitudes on induced abortion and family planning among Sri Lankan women seeking termination of pregnancy. Ceylon Med J. 2004;49(1). doi: 10.4038/cmj.v49i1.3278 15255322

[19] Abeysena C, Jayawardana P, Seneviratne R. Risk factors for spontaneous abortion. J Coll Community Physicians Sri Lanka. 2009;14(1):14.

[20] Nayana SY, Patabendige A. Effects of Selected Socio-Demographic, Maternal, Service-Related and Psychological Risk Factors for Still Births Delivered in District General Hospital, Matara, Sri Lanka During 2015–2018. Indian J Med Res Pharm Sci. 2019; 6(2):32–6.

[21] Arambepola C, Rajapaksa LC, Attygalle D, Moonasinghe L. Relationship of family formation characteristics with unsafe abortion: Is it confounded by women’s socio-economic status?—A case-control study from Sri Lanka. Reprod Health. 2016 Jun 17;13(1):1–9. doi: 10.1186/s12978-016-0173-5 27316713 PMC4912832

[22] Thalagala N. Unsafe abortions in Sri Lanka—facts and risk profile. J Coll Community Physicians Sri Lanka. 2012;15(1).

[23] Arambepola C, Rajapaksa LC. Risk of unsafe abortion associated with long-term contraception behaviour: A case control study from Sri Lanka. BMC Pregnancy Childbirth. 2017 Jun 29;17(1):1–10. 28662700 10.1186/s12884-017-1376-7 PMC5493010

[24] Arambepola C, Rajapaksa LC. Decision making on unsafe abortions in Sri Lanka: A case-control study. Reprod Health. 2014 Dec 17;11(1):1–8. 25518959 10.1186/1742-4755-11-91 PMC4280739

[25] Suchira S, De Silva W. Induced Abortion. In: Sri Lankan Youth: sexual and reproductive health. Child Fund Sri Lanka; 2020. p. 176–88.

[26] Suranga M, Silva K, Senanayake L. Factors associated with attitudes on induced abortion–A community based study among adults in Colombo City of Sri Lanka. Sri Lanka J Adv Soc Stud. 2016;6(2):55–80.

[27] Abraha D, Welu G, Berwo M, Gebretsadik M, Tsegay T, Gebreheat G, et al. Knowledge of and Utilization of Emergency Contraceptive and Its Associated Factors among Women Seeking Induced Abortion in Public Hospitals, Eastern Tigray, Ethiopia, 2017: A Cross-Sectional Study. Biomed Res Int. 2019;2019. doi: 10.1155/2019/7209274 31828125 PMC6886317

[28] Gutema RM, Dina GD. Knowledge, attitude and factors associated with induced abortion among female students ‘of Private Colleges in Ambo town, Oromia regional state, Ethiopia: a cross-sectional study. BMC Womens Health. 2022 Dec 1;22(1):1–11. 35982447 10.1186/s12905-022-01935-3 PMC9389833

[29] Wang T, Jiang Q. Recent trend and correlates of induced abortion in China: evidence from the 2017 China Fertility Survey. BMC Womens Health. 2022 Dec 1;22(1):1–16. 36434604 10.1186/s12905-022-02074-5 PMC9700931

[30] Tesfaye G, Hambisa MT, Semahegn A. Induced abortion and associated factors in health facilities of guraghe zone, southern Ethiopia. J Pregnancy. 2014;2014. doi: 10.1155/2014/295732 24800079 PMC3988865

[31] Obiyan MO, Olaleye AO, Oyinlola FF, Folayan MO. Factors associated with pregnancy and induced abortion among street-involved female adolescents in two Nigerian urban cities: a mixed-method study. BMC Health Serv Res. 2023 Dec 1;23(1):1–11. 36627625 10.1186/s12913-022-09014-x PMC9832642

[32] Rajapaksa LC. Estimates of induced abortion in urban and rural Sri Lanka. J Coll Community Physicians Sri Lanka. 2002 Dec 29;7(1):10.

[33] Udayanthi G, De Silva B, Perera H. The factors influencing miscarriage: mothers’ perspective. In: Annual Academic Sessions. Open University of Sri Lanka; 2012. p. 133–5.

[34] Kanmaz AG, Inan AH, Beyan E, Budak A. The effects of threatened abortions on pregnancy outcomes. Ginekol Pol. 2019;90(4):195–200. doi: 10.5603/GP.a2019.0035 30901073

[35] Iqbal Z, Jilanee S, Uppada L, Imtiaz S, Khan H, Shah S, et al. Evaluating the Clinical Risk Factors Associated With Miscarriages in Women in Karachi, Pakistan. Cureus. 2021;13(10):e19057. doi: 10.7759/cureus.19057 34824942 PMC8610211

[36] Alahakoon A, Ratnayake C, Karunakaran K, Tennakoon S. A Cross-Sectional Study on Maternal Factors for Stillbirths Taking Place in Hospitals in Kandy, Sri Lanka. In: Young Scientists Forum. 2022. p. 163–70.

[37] Neogi SB, Sharma J, Negandhi P, Chauhan M, Reddy S, Sethy G. Risk factors for stillbirths: How much can a responsive health system prevent? BMC Pregnancy Childbirth. 2018 Jan 18;18(1):1–10. 29347930 10.1186/s12884-018-1660-1 PMC5774063

[38] Department of Census and Statistics Sri Lanka. Demographic and Health Survey—2016. Colombo; 2017.

[39] Department of Census and Statistics Sri Lanka. Dissemination Policy on Microdata. Colombo; 2007.

[40] Shrestha N. Detecting Multicollinearity in Regression Analysis. Am J Appl Math Stat. 2020;8(2):39–42.

[41] O’Connell AA, Goldstein J, Rogers HJ, Peng CYJ. Multilevel logistic models for dichotomous and ordinal data. Multilevel Model Educ data. 2008;199–242.

[42] Fantay Gebru K, Mekonnen Haileselassie W, Haftom Temesgen A, Oumer Seid A, Afework Mulugeta B. Determinants of stunting among under-five children in Ethiopia: a multilevel mixed-effects analysis of 2016 Ethiopian demographic and health survey data. BMC Pediatr. 2019;19(1):1–13. 31153381 10.1186/s12887-019-1545-0 PMC6544992

[43] Heazell AEP, Newman L, Lean SC, Jones RL. Pregnancy outcome in mothers over the age of 35. Curr Opin Obstet Gynecol. 2018;30(6):337–43. doi: 10.1097/GCO.0000000000000494 30239372

[44] Carolan M, Nelson S. First mothering over 35 years: questioning the association of maternal age and pregnancy risk. Health Care Women Int. 2007;28(6):534–55. doi: 10.1080/07399330701334356 17578714

[45] Nybo Andersen AM, Wohlfahrt J, Christens P, Olsen J, Melbye M. Maternal age and fetal loss: population based register linkage study. BMJ. 2000 Jun 24;320(7251):1708–12. doi: 10.1136/bmj.320.7251.1708 10864550 PMC27416

[46] Jayatunge T, Kumara S, Lucas N. Advanced maternal age and perinatal outcomes in two tertiary care maternity hospitals in Colombo, Sri Lanka. Sri Lanka J Child Heal. 2022;51(2):241–8.

[47] Assessing Your Weight | Healthy Weight, Nutrition, and Physical Activity | CDC [Internet]. [cited 2023 Oct 18]. https://www.cdc.gov/healthyweight/assessing/index.html

[48] Yao R, Ananth C V, Park BY, Pereira L, Plante LA, Consortium PR, et al. Obesity and the risk of stillbirth: a population-based cohort study. Am J Obstet Gynecol. 2014;210(5):457–e1. doi: 10.1016/j.ajog.2014.01.044 24674712

[49] Metwally M, Ong KJ, Ledger WL, Li TC. Does high body mass index increase the risk of miscarriage after spontaneous and assisted conception? A meta-analysis of the evidence. Fertil Steril. 2008;90(3):714–26. doi: 10.1016/j.fertnstert.2007.07.1290 18068166

[50] Patel A, Prakash AA, Das PK, Gupta S, Pusdekar YV, Hibberd PL. Maternal anemia and underweight as determinants of pregnancy outcomes: cohort study in eastern rural Maharashtra, India. BMJ Open. 2018 Aug 1;8(8):e021623. doi: 10.1136/bmjopen-2018-021623 30093518 PMC6089300

[51] Veleva Z, Tiitinen A, Vilska S, Hydén-Granskog C, Tomás C, Martikainen H, et al. High and low BMI increase the risk of miscarriage after IVF/ICSI and FET. Hum Reprod. 2008 Apr 1;23(4):878–84. doi: 10.1093/humrep/den017 18281684

[52] Ghimire PR, Akombi-Inyang BJ, Tannous C, Agho KE. Association between obesity and miscarriage among women of reproductive age in Nepal. PLoS One. 2020 Aug 1;15(8):e0236435. doi: 10.1371/journal.pone.0236435 32760090 PMC7410243

[53] Broughton DE, Moley KH. Obesity and female infertility: potential mediators of obesity’s impact. Fertil Steril. 2017 Apr 1;107(4):840–7. doi: 10.1016/j.fertnstert.2017.01.017 28292619

[54] Boutari C, Pappas PD, Mintziori G, Nigdelis MP, Athanasiadis L, Goulis DG, et al. The effect of underweight on female and male reproduction. Metabolism. 2020 Jun 1;107:154229. doi: 10.1016/j.metabol.2020.154229 32289345

[55] Seidu A-A, Ahinkorah BO, Ameyaw EK, Hubert A, Agbemavi W, Armah-Ansah EK, et al. What has women’s reproductive health decision-making capacity and other factors got to do with pregnancy termination in sub-Saharan Africa? evidence from 27 cross-sectional surveys. PLoS One. 2020;15(7):e0235329. doi: 10.1371/journal.pone.0235329 32702035 PMC7377410

[56] Seidu A-A, Ahinkorah BO, Agbemavi W, Amu H, Bonsu F. Reproductive health decision-making capacity and pregnancy termination among Ghanaian women: Analysis of the 2014 Ghana demographic and health survey. J Public Health (Bangkok). 2021;29(1):85–94.

[57] Jellesen R, Strandberg-Larsen K, Jørgensen T, Olsen J, Thulstrup AM, Andersen A-MN. Maternal use of oral contraceptives and risk of fetal death. Paediatr Perinat Epidemiol. 2008;22(4):334–40. doi: 10.1111/j.1365-3016.2008.00942.x 18578746

[58] García-Enguídanos A, Martínez D, Calle ME, Luna S, de Bernabé JV, Domínguez-Rojas V. Long-term use of oral contraceptives increases the risk of miscarriage. Fertil Steril. 2005;83(6):1864–6. doi: 10.1016/j.fertnstert.2004.11.085 15950668

[59] Jovanovic L, Dawood MY, Landesman R, Saxena BB. Hormonal profile as a prognostic index of early threatened abortion. Am J Obstet Gynecol. 1978;130(3):274–8. 623166

[60] De Silva WI, Dayananda RA, Nishanti Perera NWPDB. Contraceptive behaviour of abortion seekers in Sri Lanka. Asian Popul Stud. 2006 Mar;2(1):3–18.

[61] Balayla J, Azoulay L, Abenhaim HA. Maternal marital status and the risk of stillbirth and infant death: a population-based cohort study on 40 million births in the United States. Women’s Heal Issues. 2011;21(5):361–5. doi: 10.1016/j.whi.2011.04.001 21689945

[62] Arntzen A, Moum T, Magnus P, Bakketeig LS. Marital status as a risk factor for fetal and infant mortality. Scand J Soc Med. 1996;24(1):36–42. doi: 10.1177/140349489602400106 8740874

[63] Anon. Understanding Abortion in Sri Lanka: An Attitudinal Study. Colombo; 2023.

[64] Katz VL. Work and work-related stress in pregnancy. Clin Obstet Gynecol. 2012 Sep;55(3):765–73. doi: 10.1097/GRF.0b013e318253b192 22828109

[65] Jansen PW, Tiemeier H, Verhulst FC, Burdorf A, Jaddoe VWV, Hofman A, et al. Employment status and the risk of pregnancy complications: the Generation R Study. Occup Environ Med. 2010 Jun 1;67(6):387–94. doi: 10.1136/oem.2009.046300 19955575

[66] Khojasteh F, Arbabisarjou A, Boryri T, Safarzadeh A, Pourkahkhaei M. The Relationship between Maternal Employment Status and Pregnancy Outcomes. Glob J Health Sci. 2016 Dec 18;8(9):37. doi: 10.5539/gjhs.v8n9p37 27157153 PMC5064079

[67] Wickramagamage C. “Her Body, Her Right"?: Interrogating the Discourse on Abortion in Sri Lanka. Sri Lanka J Soc Sci. 2004;27(1&2):17–59.

[68] Anon. New brief outlines devastating harms from tobacco use and exposure to second-hand tobacco smoke during pregnancy and throughout childhood—Report calls for protective policies [Internet]. World Health Organization. 2021. https://www.who.int/news/item/16-03-2021-new-brief-outlines-devastating-harms-from-tobacco-use-and-exposure-to-second-hand-tobacco-smoke-during-pregnancy-and-throughout-childhood

[69] Pineles BL, Park E, Samet JM. Systematic Review and Meta-Analysis of Miscarriage and Maternal Exposure to Tobacco Smoke During Pregnancy. Am J Epidemiol. 2014 Apr 4;179(7):807. doi: 10.1093/aje/kwt334 24518810 PMC3969532

[70] Hyland A, Piazza KM, Hovey KM, Ockene JK, Andrews CA, Rivard C, et al. Associations of lifetime active and passive smoking with spontaneous abortion, stillbirth and tubal ectopic pregnancy: a cross-sectional analysis of historical data from the Women’s Health Initiative. Tob Control. 2015 Jul 1;24(4):328–35. doi: 10.1136/tobaccocontrol-2013-051458 24572626

[71] Lin S, Li J, Zhang Y, Song X, Chen G, Pei L. Maternal Passive Smoking, Vitamin D Deficiency and Risk of Spontaneous Abortion. Nutrients. 2022 Sep 1;14(18):3674. doi: 10.3390/nu14183674 36145050 PMC9501103

[72] Míguez MC, Pereira B. Effects of active and/or passive smoking during pregnancy and the postpartum period. An Pediatría (English Ed. 2021 Oct 1;95(4):222–32. doi: 10.1016/j.anpede.2020.07.021 34556446

[73] Adibelli D, Kirca N. The relationship between gestational active and passive smoking and early postpartum complications. J Matern Neonatal Med. 2020 Jul 17;33(14):2473–9. doi: 10.1080/14767058.2020.1763294 32393083

[74] Nur N. Association between domestic violence and miscarriage: a population-based cross-sectional study among women of childbearing ages, Sivas, Turkey. Women Heal. 2014;54(5):425–38. doi: 10.1080/03630242.2014.897676 24795047

[75] Afiaz A, Biswas RK, Shamma R, Ananna N. Intimate partner violence (IPV) with miscarriages, stillbirths and abortions: Identifying vulnerable households for women in Bangladesh. PLoS One. 2020;15(7):e0236670. doi: 10.1371/journal.pone.0236670 32722708 PMC7386588

[76] Cokkinides VE, Coker AL, Sanderson M, Addy C, Bethea LESA. Physical violence during pregnancy: maternal complications and birth outcomes. Obstet Gynecol. 1999 May 1;93(5):661–6. doi: 10.1016/s0029-7844(98)00486-4 10912963

[77] Morland LA, Leskin GA, Rebecca Block C, Campbell JC, Friedman MJ. Intimate Partner Violence and Miscarriage. J Interpers Violence. 2008 Feb 13;23(5):652–69. 18272727 10.1177/0886260507313533

[78] Senanayake L. Domestic violence: an emerging concern in maternity care. Sri Lanka J Obstet Gynaecol. 2012 Oct 25;33(4):142.

[79] Anon. Census of Population and Housing Sri Lanka 2012. Colombo; 2015.

[80] Thalagala N. Process determinants, and impact of unsafe abortions in Sri Lanka. Family Planning Association, Sri Lanka; 2010. 83 p.

